# Forming a medicines pricing policy for low and middle-income countries (LMICs): the case for Pakistan

**DOI:** 10.1186/s40545-022-00413-3

**Published:** 2022-02-24

**Authors:** Zaheer-Ud-Din Babar

**Affiliations:** Department of Pharmacy, University of Huddersfield, Queensgate, Huddersfield, HD1 3DH UK

**Keywords:** Low and middle income countries (LMICs), Pakistan, Medicine prices, Access to medicines

## Abstract

Equitable access to medicines has played a vital role to improve patient health outcomes and reducing mortality globally. However, it is important to note that medicines pricing is a key determinant in promoting access to medicines. The studies and empirical data have shown that there are wide variations in prices across countries for the same brand of medicines. World Health Organisation (WHO) has provided guidelines to formulate country pharmaceutical pricing policies. However, little is known how these guidelines will be used in the country-specific setting. This commentary provides guiding principles and outlines the basis to form a medicines pricing policy in a low and middle-income country, Pakistan. It discusses the current medicines pricing policy and provides suggestions for future work. The suggested medicines pricing structure and lessons learned in this commentary can also be applied in other low-resource settings.

## Introduction

Access to medicines is a fundamental human right and medicines pricing is a key factor, which determines it [1, 2]. The United Nations Sustainable Development Goals (SDGs) 3.8 deals with access and it states “financial risk protection, access to quality essential healthcare services and access to safe, effective, quality and *affordable* essential medicines and vaccines for all” [3].

Equitable access to medicines has played a vital role to improve patient health outcomes and reducing mortality. For example, cardiovascular disease, (disability-adjusted life-years per 100,000 people) fell by 30% in rich countries between 1990 and 2017, however, it declined by only 9% in poor countries [4, 5]. This could be linked with medicines inequity, as the World Health Organization estimated that one-third of the world’s population does not have access to essential medicines [6]. Major barriers to access include the non-availability of medicines and high medicine prices.

Most countries in the world control medicine prices either through direct control (controlling prices at the government level) or through indirect control (through using pharmacoeconomic ways). The studies have shown that in the countries where the prices are controlled, affordability is better [7–10].

Forming a medicines pricing policy in a country is a challenging task. This is because the countries are at a different level of the health system (b) western developed health systems are used as guidance where pharmaceutical systems are inherently different from their own (c) policymakers need to understand the pharmacy and reimbursement system of their own country before setting-up a medicines pricing policy.

This commentary provides guiding principles and outlines the basis to form a medicines pricing policy in a low and middle-income country, Pakistan. It provides a critique of the previous medicines pricing policy in the country and provides suggestions for future work. However, the suggested medicines pricing structure and lessons learned in this commentary can also be applied in other low-resource settings. The sections in this commentary include (1) Key Issues to Understand Global Medicines Pricing Policies (2) Pakistan’s Medicines Pricing Policy 2018 (3) Pricing Policies for Pakistan (4) Generic Pricing Structure in Pakistan (5) Recommendations for Future Work.

### Objectives of the medicines pricing policy

The medicines pricing policy of a country needs to cover the following objectives: (a) to ensure access to effective, safe and quality assured affordable medicines for all (b) to establish fair medicines pricing mechanisms that can improve access, (c) to support the pharmaceutical industry. However, before setting up a medicines pricing policy in an individual country, it is vital to understand the global medicines pricing setup.

## Understanding global medicines pricing policies

Understanding global medicines pricing structure is vital before discussing medicines pricing structure in a low and middle-income country (LMICs). In most western developed countries including the United Kingdom, Australia, and New Zealand, there is a public subsidy for pharmaceuticals in both public sector hospitals as well as at private-sector retail pharmacies. The governments subsidize the cost of medicines, and as a result, the private out-of-pocket expenditures for consumers are low hence medicines pricing does not impact consumers, the way it does in countries where out-of-pocket expenses are high.

The prices in high-income developed countries are negotiated and controlled through specific drug buying agencies. These agencies are set up separately from drug regulatory authorities. For example, in each of the following countries, there are two separate agencies, one is to ensure drug safety, effectiveness, and quality and the other for cost effectiveness of medicines. For example, in New Zealand; the Medsafe [11] is the regulator and the Pharmaceutical Management Agency of NZ (PHARMAC) [12] is responsible for access and cost effectiveness. In the UK, The Medicines and Healthcare products Regulatory Agency (MHRA) [13] is the regulator whereas National Institute for Health and Care Excellence (NICE) [14] considers the value of new medicines based on the cost-effectiveness of medicines/ and Quality Adjusted Life Years (QALYs).

There are only a few countries in the world, where medicine prices are not controlled. The prices in these countries are very high; examples include the United States and Malaysia [7] South Asia (Pakistan, India, Bangladesh) [8–10, 15] controls medicine prices at the consumer level and private sector retail pharmacies. For the same (an individual brand,) prices are uniform and fixed. This system has generated low prices in international terms for Pakistan, India, Bangladesh [8–10, 15].

## Pakistan’s medicines pricing policy 2018

In Pakistan, there are significant out-of-pocket costs for pharmaceuticals for consumers and patients. The prices are controlled at the retail pharmacy level and the medicines can’t be sold above the Maximum Retail Price (MRP). The MRP is written on the medicines package. These are set uniformly throughout the country and are set by the Ministry of National Health Services, Regulation and Coordination, Government of Pakistan.

An analysis was also performed on Pakistan’s Pakistan’s Medicines Pricing Policy 2018 [16]. Though several medicines are reasonably priced in international terms [9, 10], however, there are others for which the prices are high. The majority of these high-priced medicines are innovator brands, though some of them are generics. This has been observed in several empirical studies conducted in the Pakistani context [9, 10].The use of a cost-plus pricing formula has worked for Pakistan in the past to keep pricing affordable for many medicines. However, with the emerging innovative medicines, this formula is getting obsolete, and the WHO has advised against using this formula [17].

Hence, it is critical in this context to analyze and explore prices of medicines case-by-case basis rather than applying a blanket formula. This is critical specifically for innovator brands. Several countries use medicine prices from other countries as a benchmark. For example, Pakistan’s 2018 medicines pricing policy measures the average retail price of a basket of countries including, Sri Lanka, Indonesia, Malaysia, Bangladesh, and Lebanon. Though prices in Sri Lanka are low [18] however other benchmark countries including Malaysia, [7] Indonesia, [19] Thailand [20], and the Philippines [21] have some of the highest prices in international terms. Malaysia practices a free market economy with no control over drug prices. This has resulted in very high drug prices in the country [7]. Though Bangladesh has reasonable medicine prices, this also needs to be looked at a case-to-case basis as a recent study also showed high prices for some innovator brands in Bangladesh [8].

The medicines pricing policy 2018 also uses the average procurement prices of the Australian Pharmaceutical Benefits Scheme (PBS) [22], UK’s Drug Tariff prices [23], as a benchmark. The inclusion of these countries as a benchmark for Pakistan is challenging because (i) Australia, and the UK all practices Universal Health Coverage with part charges from consumers and no out-of-pocket payments from the marginalized sections of society. On the other hand, Pakistan has significant out-of-pocket consumers’ payments (ii) Another global challenges with the drug prices are that the listed prices from Australian PBS, and the UK’s Drug Tariff, in the public domain publications may not be actual prices. The published prices could be discounted prices after rebates by pharmaceutical companies [24] (iii) Australia has high prices for both innovator brands as well as generics [25].

## Building evidence-informed medicines pricing policies

The best way to form a pricing policy in an LMIC is by taking into account the data obtained from studies using WHO/HAI pricing methodology [26]. The pricing research has been done in over 50 countries by using this methodology. Through WHO/HAI surveys, the prices and availability of innovator brands and lowest-priced generics are measured. It provides room for profits for the pharmaceutical industry and at the same time, it advocates a rational pricing structure to promote affordability. The methodology compares the median community retail pharmacy prices/public procurement prices with the International reference prices (IRPs) obtained from the Management Sciences for Health (MSH), USA [27]. By comparing IRPs with median community retail pharmacy prices, a Median Price Ratio (MPR) is obtained.

An MPR could be used as a guide to benchmark prices in a Low to Middle Income Country (LMIC), how high or low are /or should be in a given context. For example, a median price ratio of about 1 is considered reasonable in the public sector while a ratio of roughly 3 is considered fine in the private sector [7]. It considers taxes mark-ups, profit margins, and enough margins for pharmaceutical industry growth, etc. Recent studies have shown that though the majority of medicines in Pakistan are rationally priced, the prices of some innovator brands were high when compared with the international reference prices (IRPs). For example, Acyclovir and atorvastatin were 18 times higher than IRP, Ciprofloxacin, Ceftriaxone injection, Diclofenac sodium were found 15 times higher, Fluconazole (60 times higher), Omeprazole and Simvastatin were found to be more than 12 times higher than IRPs [9, 10]. These are examples, and there may be other medicines that could be high priced. These medicines and others are commonly used by Pakistanis and this is resulting in significant out-of-pocket payments, impacting affordability. An extensive and detailed exploratory study is further needed to scope out these medicines.

## Medicines pricing policies for Pakistan

There is a need to use multiple pricing policies to achieve low prices for medicines in low and middle-income countries. The World Health Organisation has also recommended this approach [17, 28]. These policies include internal reference pricing, external reference pricing, value-based pricing, and mark-up regulations. Though WHO advises against the use of cost-plus pricing, [17] however, if this is needed then all costs and prices need to be transparent. Table 1 elaborates suggested pharmaceutical pricing policies for Pakistan. Many of these are adopted from the WHO guidelines on country pharmaceutical pricing policies [28].

**Table 1:** A snapshot of suggestive pharmaceutical pricing policies for Pakistan

No	Pricing Policies	Suggested in Pakistan	Mark-ups/percentage	Global Practices
1	Internal Reference Pricing	Yes	I.Pakistan, the first generic medicine should be priced 40–50% below the originator, whereas the price of the first biosimilar had to be only 30% lower than the reference product II.In the case of “Me-too” medicines, a cost-minimization analysis should be done III.For the new presentation in the same dosage of medicine already marketed. The price should be worked out based on the arithmetic mean of the prices of previously launched medicines [12] IV.For the presentation of different dosages of medicine already marketed. The price must not exceed the average price, weighted by sales, of available presentations of the medicine that have the same active ingredients, strength, and dosage form	Varies from one country to another In Brazil Generic medicine price can’t exceeds 65% of the respective reference medicine [37] In the Czech Republic the first generic medicine had to be priced 32% below the originator, whereas the price of the first biosimilar had to be 15% lower than the reference product [38]
2	External Reference Pricing	Reference countries *India, Bangladesh, Sri Lanka, South Africa, Iran, Saudi Arabia, and New Zealand*		Yes ( different countries adapt different practices)
3	Value Based Pricing	Highly specialised unit is required for implementation		Yes
4	Mark-up regulations	Yes	Currently a mark-up of 35 and 15% is applied to the wholesaler and the retailer (to be applied case to case basis) Need to develop a regressive mark-up structure, in which the mark-up rate decreases as the price increases (rather than a fixed percentage mark-up for all prices)	
5	Cost plus pricing	Difficult to understand current pricing formula. Need clarity and transparency on costs		Not used globally

### Cost-plus pricing policy

The cost-plus pricing requires significant technical and human resources. The most challenging part of cost-plus pricing is obtaining and validating information on different cost components. This needs more clarity and transparency [17]. The lack of transparency in expenses on pharmaceutical marketing also makes it challenging. There is limited evidence about the implementation and outcomes of cost-plus pricing. The countries identified to use cost-plus pricing are Vietnam, China, Sri Lanka, Bangladesh, Iran, and Pakistan. India and Colombia has discontinued applying this pricing method [17, 28–30]. In the past European Countries have used a cost-plus pricing policy, however, no European country uses this policy anymore [30]. If the cost-plus formula needs to be used in an LMIC, it has to be transparent in terms of it’s working, calculation, and different components.

### External price referencing (EPR)

An increasing number of countries are using external price referencing (EPR) [31]. Using EPR, governments set medicine prices in their country based on the prices of the same medicines in other countries. External Price Referencing (EPR) is commonly used for new medicines, and evidence suggests that external reference pricing is likely to reduce the prices of medicines [31]. To implement this approach, reference prices can be obtained from verifiable data sources. It is also imperative that reference prices take into account all forms of discounts, rebates, and taxes [17–31]. It is recommended to apply EPR at the level of the ex-factory price level [31]. Pakistan can use and apply external reference pricing by benchmarking with countries such as India, Bangladesh, Sri Lanka, South Africa, Iran and New Zealand. India, Bangladesh, and Sri Lanka are all South Asian countries with similar socio-economic parameters. Saudi Arabia is listed as an early adopter of the new medicines. Iran and South Africa are countries with developing health systems, while New Zealand is a world leader in cost-effective pharmaceutical pricing.

### Internal reference pricing

Internal reference pricing is used for linking the prices of substitutable medicines, for example generic, biosimilar, or therapeutically equivalent medicines [17]. A specific type of internal price referencing is called a generic price link [31]. This policy refers to the practice of setting the price of a generic in relationship to the originator medicine, usually at a certain percentage lower than the originator price. Internal reference pricing is also specifically useful in the absence of demand-side measures to encourage the preferential prescribing and dispensing of multiple sourced medicines versus originator brands. In Pakistan, the first generic medicine could be priced 40–50% below the originator, whereas the price of the first biosimilar can be reduced to 30% lower than the reference medicine. However, these are suggestions and other pricing models can also be explored. For example, in Austria, greater pricing reductions have been seen in the case of generic medicines [32]. Similarly, successful drug switching can be seen for statins in the Netherlands [33].

### Value-based pricing

Value-based pricing uses health technology assessment (HTA) and it must include an analysis on budget impact and affordability from the perspective of the payers and the patients^.^[17, 29] Conducting value-based pricing is a highly specialized task and needs a health economics unit together with input from pharmacists, clinicians, evidence-based practitioners etc. Adequate resources and skilled personnel are needed to implement value-based pricing in low and middle-income countries [34]. Value-based pricing can specifically be used for high-cost medicines and innovator brands [30]. Pakistan can take a gradual stepwise approach to develop legislative and technical capacity to implement value-based pricing using Health Technology Assessment (HTA). The country needs to produce indigenous data to strengthen this highly specialized field by improving and promoting research and training in this area.

However, it is imperative to note that the use of value-based pricing is questioned in Low and Middle-Income Countries, as it is of little use if the essential medicines are not available and affordable for masses within a country. The lessons can also be learned from high-income countries. For example, Stockholm in Sweden has successfully operated a limited list of 2000 essential medicines covering over 90% of the needs in ambulatory care [35, 36]. This approach has been very effective to promote the rationale use of medicines. This also begs the question that why do LMICs need new high-priced medicines when patients in ambulatory setting can be well treated with a smaller list of medicines.

### Mark-up regulations

Currently, a mark-up of 35 and 15% is applied to the wholesaler and the retailer, in Pakistan respectively. This is fine for medicines that are rationally priced. However, there is a need to develop a regressive mark-up structure, in which the mark-up rate decreases as the price increases. This needs to be implemented rather than a fixed percentage mark-up for all prices. It is suggested by the WHO to use remuneration and mark-up regulation as incentives for supplying specific medicines such as generic medicines, low-volume medicines, as well as orphan drugs [17]. Transparency is needed when setting up mark-ups along the supply and distribution chain, including disclosure of any rebates and discounts. There is a need to encourage the early market entry of generic and biosimilar medicines through legislative and administrative reforms [17, 30].

### Summary-proposed medicines pricing policy structure for Pakistan

Table 1 provides a snapshot of suggested pharmaceutical pricing policies for Pakistan. For innovator brands, the price should take into account the use of the external reference pricing (EPR) system, and preferably the price must not exceed the medicine’s lowest price in any of the following countries; Saudi Arabia, South Africa, Bangladesh, Iran, India, and Sri Lanka. For “Me-too” medicines, a cost-minimization analysis should be encouraged [37].

For the new presentation in the same dosage of medicine already marketed. The price should be worked out based on the arithmetic mean of the prices of previously launched medicines [37]. It is suggested that the price must not exceed the average price, weighted by sales, of available presentations of the medicine that have the same active ingredients, strength, and dosage form [37]. Generic medicine should not exceed 40–50% of the respective reference medicine.

## Recommendations for the future

The decision to formulate and set prices needs to be based on empirical pricing studies, hence ongoing research is needed in LMICs in this context. Though WHO has provided guidelines to form country pharmaceutical pricing policies, however translating them into local setting is a complex task. Adequate human resources and skilled personnel are required for implementation, monitoring, and ongoing evaluation of medicines prices. A center for evidence-based medicines and pharmacoeconomics should be established within the country. Pakistan also needs to undertake a regular price revision at the pre-specified frequency when using external reference pricing.

## References

[1] World Health Organization, Health action international (2003) medicine prices—a new approach to measurement. Geneva: World Health Organization. http://whqlibdoc.who.int/hq/2003/WHO_EDM_PAR_2003.2.pdf. Accessed 4 Nov 2021.

[2] The United Nations Secretary-General’s High-Level Panel on Access to Medicines Report, September 14, 2016

[3] UN Sustainable development goals. https://sdgs.un.org/goals. Accessed 4 Nov 2021.

[4] Nolte E, McKee M (2004). Does health care save lives? Avoidable mortality revisited.

[5] IHME. GBD Compare. 2019. https://vizhub.healthdata.org/gbd-compare/. Accessed 4 Nov 2021.

[6] Hogerzeil HV, Mirza Z (2011). The world medicines situation 2011: access to essential medicines as part of the right to health.

[7] Babar ZUD, Ibrahim MIM, Singh H, Bukahri NI, Creese A (2007). Evaluating drug prices, availability, affordability, and price components: implications for access to drugs in Malaysia. PLoS Med.

[8] Kasonde L, Tordrup D, Naheed AB, ZUD.  (2019). Evaluating medicine prices, availability and affordability in Bangladesh using World Health Organisation and Health Action International methodology. BMC Health Serv Res.

[9] Saeed A, Saeed H, Saleem Z, Babar ZUD (2020). Impact of National Drug Pricing Policy 2018 on access to medicines in Lahore division, Pakistan: a pre-post survey study using WHO/HAI methodology. BMJ Open.

[10] Saeed A, Saeed H, Saleem Z, Fang Y, Babar Z-U-D (2019). Evaluation of prices, availability and affordability of essential medicines in Lahore Division, Pakistan: a cross-sectional survey using WHO/HAI methodology. PLoS ONE.

[11] New Zealand Medicine safety agency. https://www.medsafe.govt.nz/. Accessed 4th Nov 2021.

[12] Pharmaceutical Management Agency of New Zealand. https://pharmac.govt.nz/. Accessed 4th Nov 2021.

[13] National Institute for Health and Care Excellence. https://www.nice.org.uk/. Accessed 4th Nov 2021.

[14] Medicines and Healthcare Products Regulatory Agency. https://www.gov.uk/government/organisations/medicines-and-healthcare-products-regulatory-agency. Accessed 4th November 2021

[15] Kotwani A, Ewen M, Dey D (2007). Medicine prices and availability at six sites in India: using the WHO-HAI methodology. Ind J Med Res.

[16] Drug Pricing Policy 2018, The Gazette of Pakistan, Drug Regulatory Authority of Pakistan, Ministry of National Health Services, Regulations and Coordination, Government of Pakistan, Islamabad June 12, 2018.

[17] WHO guideline on country pharmaceutical pricing policies, second edition. Geneva: World Health Organization; 2020. Licence: CC BY-NC-SA 3.0 IGO. 33950613

[18] Senarathna SM, Mannapperuma U, Fernandopulle BM (2011). Medicine prices, availability and affordability in Sri Lanka. Indian J Pharmacol.

[19] Wasir R, Irawati S, Makady A, Postma M, Goettsch W, Buskens E (2019). Use of medicine pricing and reimbursement policies for universal health coverage in Indonesia. PLoS ONE.

[20] Sooksriwong C, Yoongthong W, Suwattanapreeda S, Chanjaruporn F (2009). Medicine prices in Thailand: a result of no medicine pricing policy. Southern Med Rev.

[21] Lambojon K, Chang J, Saeed A, Hayat K, Li P, Jiang M, Atif N, Desalegn GK, Khan FU, Fang Y (2020). Prices, availability and affordability of medicines with value-added tax exemption: a cross-sectional survey in the philippines. Int J Environ Res Public Health.

[22] Australian Pharmaceutical Benefits Scheme https://www.pbs.gov.au/info/about-the-pbs. Accessed 4 Nov 2021.

[23] UK Drug Tariff. https://www.nhsbsa.nhs.uk/pharmacies-gp-practices-and-appliance-contractors/drug-tariff. Accessed 4 Nov 2021.

[24] Vogler S, Paris V, Ferrario A, Wirtz VJ, de Joncheere K, Schneider P, Pedersen HB, Dedet G, Babar ZU (2017). How can pricing and reimbursement policies improve affordable access to medicines? Lessons learned from european countries. Appl Health Econ Health Policy.

[25] Vitry A, Shute R, Din Babar ZU (2018). Access to high-cost medicines in Australia. Equitable access to high-cost pharmaceuticals.

[26] WHO. Measuring medicine prices, availability, affordability and price components 2nd edition: http://haiweb.org/publication/measuring-medicine-pricesavailability-affordability-and-price-components-2nd-ed/. Accessed 4 Nov 2021.

[27] MSH. International Drug Price Indicator Guide [Internet]. Management Sciences for Health. 2017 . https://www.msh.org/resources/international-drug-price-indicator. Accessed 4 Nov 2021.

[28] World Health Organization. WHO guideline on country pharmaceutical pricing policies. Geneva. 2013. http://apps.who.int/medicinedocs/documents/s21016en/s21016en.pdf. Accessed 4 Nov 2021. 25473702

[29] Nguyen TA, Knight R, Roughead EE, Brooks G, Mant A (2014). Policy options for pharmaceutical pricing and purchasing: issues for low-and middle-income countries. Health Policy Plan.

[30] Vogler S (2018). Medicine price surveys, analyses and comparisons: evidence and methodology guidance.

[31] Vogler S, Vogler SM (2019). Assessment of External Price Referencing and Alternative Policies. Medicine price surveys, analyses and comparisons: evidence, methodology and guidance.

[32] Martikainen JE, Maljanen T, Koskinen H, Vogler S (2015). Impact of generic price linkage system and reference price system on prices of pharmaceuticals–comparison of Austria and Finland. J Pharm Policy Pract.

[33] Glerum PJ, Maliepaard M, de Valk V, Burger DM, Neef K (2020). Drug switching in the Netherlands: a cohort study of 20 active substances. BMC Health Serv Res.

[34] Babar ZU, Scahill S (2010). Is there a role for pharmacoeconomics in developing countries?. Pharmacoeconomics.

[35] Gustafsson LL, Wettermark B, Godman B, Andersén-Karlsson E, Bergman U, Hasselström J, Hensjö LO, Hjemdahl P, Jägre I, Julander M, Ringertz B, Schmidt D, Sjöberg S, Sjöqvist F, Stiller CO, Törnqvist E, Tryselius R, Vitols S, von Bahr C, Regional Drug Expert Consortium (2011). The 'wise list'- a comprehensive concept to select, communicate and achieve adherence to recommendations of essential drugs in ambulatory care in Stockholm. Basic Clin Pharmacol Toxicol.

[36] Eriksen J, Gustafsson LL, Ateva K, Bastholm-Rahmner P, Ovesjö ML, Jirlow M, Juhasz-Haverinen M, Lärfars G, Malmström RE, Wettermark B, Andersén-Karlsson E, Stockholm DTC (2017). High adherence to the 'Wise List' treatment recommendations in Stockholm: a 15-year retrospective review of a multifaceted approach promoting rational use of medicines. BMJ Open.

[37] Balbino JE, Vogler SM (2019). Medicine Prices in Latin American Countries. Medicine price surveys, analyses and comparisons: evidence, methodology and guidance.

[38] Vogler S, Schneider P (2017). Do pricing and usage-enhancing policies differ between biosimilars and generics? Findings from an international survey. GaBI J.

